# Physicians' migration in Europe: an overview of the current situation

**DOI:** 10.1186/1472-6963-7-201

**Published:** 2007-12-10

**Authors:** Miguel A García-Pérez, Carlos Amaya, Ángel Otero

**Affiliations:** 1 Department of Preventive Medicine and Public Health. School of Medicine, Universidad Autónoma. Madrid, Spain; 2 Department of Studies and Research, Fundación CESM. Madrid, Spain

## Abstract

**Background:**

The migration of medical professionals as a result of the expansion of the European Union is cause for concern. But there is a significant lack of information available about this phenomenon.

**Methods:**

Search of secondary databases about the presence of european doctors working abroad, through two search engines in the Internet (Google and Pubmed) and a survey of professional organisations and regulators in countries of the European Union.

**Results:**

The United Kingdom has more foreign doctors than all other European countries for which figures are available (Ireland, France, Germany, Norway, Portugal, Italy, Austria and Poland). Some 74,031 foreign doctors are registered in the UK, 30.94% of the total. European countries with the highest percentage of doctors working abroad are Ireland (47.5%, or 10,065 doctors) and Malta (23.1%, 376 doctors). The data obtained from Norway, France and Germany do not indicate an increase in the migration of professionals from countries recently incorporated into the EU.

**Conclusion:**

There is significant mobility and heterogeneous distribution of doctors within the EEA and it should be cause for concern among health care authorities. However, there is no evidence about a possible increase in this phenomenon after the recent expansion of the EU.

## Background

A key factor in providing medical care is the availability of qualified and motivated health care professionals [1-4]. However, a extended perception exists in Europe that there is an inadequate provision and/or a poor distribution of them [5-7].

The current process of political and economic globalisation, in which European legislation on free movement of persons take place, has caused significant migratory tensions. Developed countries generally act as polls of attraction for medical professionals resulting in the risk of a "brain drain" or loss of professional capital in less developed countries [1,8-11]. The recent expansion of the European Union (EU) has caused concern among authorities in recently integrated countries about the loss of professionals, although figures for previous expansions do not seem to support this concern [9,12,13].

There is, however, a significant lack of information. Existing analyses of the migration of medical personnel within the EU is almost exclusively based on data from the United Kingdom (UK). While European institutions establish reliable mechanisms to collect data, an analysis of existing data can provide valuable insights [4,14]. The purpose of this work is to gather available information and provide an initial evaluation of migratory flows of medical professionals within Europe taking in account the incomplete nature of available information.

## Methods

This work analyses mobility of doctors in the European Economic Area (EEA: European Union plus some of the non-EU member states in the European Free Trade Association: Norway, Liechtenstein, Switzerland and Iceland) using existing information in medical literature, professional organisations and/or professional regulators of the different states. Given that there are no common criteria for registration, we will refer to registered doctors (total number of registered doctors), qualified-to-practise doctors (doctors who meet some requirement to practise medicine, either qualifications, age, etc.) or practising doctors. Country of origin will be either the country where medical training was received (foreign-trained doctors) or the nationality (foreign doctors), according to available information in each case.

Two ways for getting information were used:

• A search in the Internet, both general (using the Pubmed and Google search engines, with the terms "doctor", "physician," "foreign," "emigration" and "Europe," in various combinations, and for data after the year 2000) and specific (in web pages of various professional organisations within the EU). From professional organisations, data were found for Germany (registered foreign doctors in practice, 2000–2005) [15], Italy (registered foreign doctors and odontologists, 2004) [16], Poland (practising foreign doctors, 2002) [17], France (practising foreign doctors, 2002–2005) [18], Norway (registered foreign doctors under the age of 67, 2001–2006) [19] and Ireland (foreign-trained doctors with full registration) [20]. Two additional sources were found through de Pubmed search that provide partial information for the year 2001 for Austria, Belgium, Denmark, the UK, Norway and Switzerland [11], and for the UK (2002, foreign doctors according to country where trained) and two countries outside the EU, such as Canada (2002) and the United States (2004) (international medical graduates in training or practice in both cases) [21]. There are also partial data on the presence of foreign doctors in US for the year 2006 in the webpage of the American Medical Association (AMA), although only for the twenty top-countries of origin [22]. In order to complete this information, we asked the AMA, who sent us the whole list of international medical graduates registered in the AMA masterfile in September 2006. For each country, we selected the more recent information about foreign doctors.

• A survey of professional medical organisations within the EU countries, which are members of the Standing Committee of European Doctors (SCDE – CPME) or the World Medical Association (WMA), requesting available information regarding the total number of doctors, licensed and practising doctors, doctors of foreign origin or those trained abroad (specifying countries within the EU25, European non-members, Asia, Africa, America and Oceania). We also requested data about doctors in training from other countries and the number of doctors who may are training or practising abroad. It was answered by Austrian (2005) and Portuguese (2005) organisations which provided quantitative data about legally practising foreign doctors; the UK (2005) derived us to the General Medical Council, who facilitated data about training- countries of doctors registered in it; finally, Hungary (2004) and Sweden (2006) provided approximate data and qualitative evaluations about countries and regions of origin of registered foreign doctors. Then, a 20% response rate was obtained from the survey.

Data were collected during the second quarter of 2005 (except for Swedish and US's data, received in February and December 2006 respectively) and then tabulated according to the following indicators:

 number of foreign doctors (in whole and by country of origin) and percentage over the total number of physicians in the destination countries where data were available;

 number of doctors registered abroad for each European country;

*emigration factor *for each European country (considered as the relation between doctors practising abroad and the total number of doctors related to that country -practising in it or abroad-) [21,23].

 average annual variation of doctors from European countries where temporal series were available (Germany, France and Norway), to estimate possible repercussions of EU expansion on professional migration within the EEA.

Given the recent incorporation of Rumania and Bulgaria to the EU, we show results for those countries included in the group of the new members (EU+12), all of them joined after the year 2004, although data collection refers always to periods prior to their adhesion.

## Results

**Figure 1:**
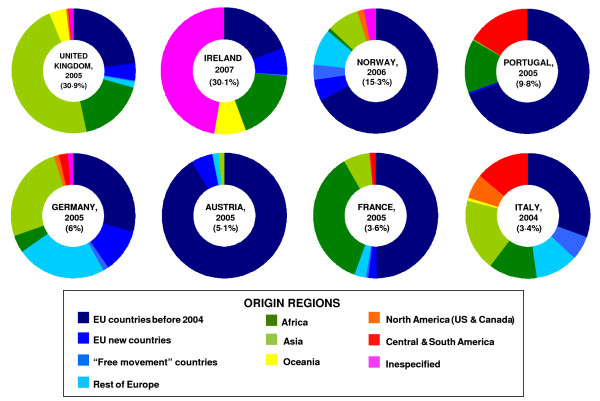
**Foreign (-trained) physicians and their distribution by origin regions in eight EEA countries**. Percentage besides the country name show the proportion of foreign (trained) physicians in that country.

Table 1 offers data obtained in our study according to the number of foreign doctors (with special attention to doctors of European origin) practising within the EEA. Figures show that the UK has more foreign doctors than all other countries included in the table. Figure 1 shows the distribution of foreign doctors according to their geographic origin.

**Table 1:** Foreign (-trained) physicians in eight EEA-countries and their geopolitical region of origin (with a special interest on European physicians).

		DESTINATION COUNTRY*			
		
		**UK, 2005**	**IRE, 2007**	**GERM, 2005**	**ITA, 2004**
**ACTIVE/REGISTERED PHYSICIANS ***Total (ratio per 100,000 inhabitants)*		**239,274 (398.4)**	**15,512 (356.3)**^†^	**307,577 (372.8)**	**365,652 (631.7)**
**FOREIGN PHYSICIANS ^†^**		**74,031**	**4,663**	**18,582**	**12,525**
SOURCE COUNTRY/REGION*					
**EU**		**20,863**	**1,224**	**8,611**	**3,829**
***EU 15*,**		***17,085***	***893***	***5,394***	
	*mainly from*	*IRE (5,959) GERM (4,026)*	*UK (592) GER (103)*	*GRE (1,357) AUS (1,269)*	*GERM (1,034) FRA (649) GRE (649)*
***EU+12*,**		***3,778***	***331***	***3,217***	
	*mainly from*	*POL (1,424) HUN (647)*	*POL (155) HUN (59)*	*POL (1,171) ROM (692)*	*ROM (389) POL (207)*
**OTHER IN EUROPE**		**982**		**3,509**	**1,351**
***Non-EU FMC *^§^,**		***222***	***9***		
	*mainly from*	*SWZ (123)*	*SWZ (7)*	*SWZ (153)*	*SWZ (760)*
***Rest of Europe***		***760***			
	*mainly from*	*RUS (342) UKR (145)*		*RUS (1,572) UKR (730)*	*SERB (437) ALB (204)*
**NORTH AMERICA (US & CAN.)**		**344**	**2**	**235**	**771**
**CENTRAL & SOUTH AMERICA**		**270**		**420**	**1,753**
**OCEANIA**		**3,290**	**378**	**15**	**107**
**AFRICA**		**12,630**	**840**	**813**	**1,590**
**ASIA**		**34,816**		**4,723**	**2,328**
**NO SPECIFIED**		**836**	**2,210**	**256**	**107**

		DESTINATION COUNTRY*			
		
		**AUS, 2005**	**PORT, 2005**	**FRA, 2005**	**NW, 2006**
**ACTIVE/REGISTERED PHYSICIANS ***Total (ratio per 100,000 inhabitants)*		**28,496 (347.2)**	**32,552 (309.2)**	**212,972 (340.7)**	**18,280 (393.9)**
**FOREIGN PHYSICIANS**^‡^		**1,442**	**3,199**	**7,665**	**2,799**
SOURCE COUNTRY/REGION*					
**EU**		**1,396**	**2,134**	**4,149**	**2,070**
***EU 15*,**		***1,317***	***2,123***	***3,811***	***1,882***
	*mainly from*	*GERM (1,003) IT (220)*	*SP (1,874) GERM (104)*	*BELG (1,368) GERM (879)*	*GERM (673) SWE (530)*
***EU+12*,**		***79***	***11***	***338***	***188***
	*mainly from*	*HUN (29) CZE (23)*	*POL (5) CZE (3)*	*ROM (158) POL (95)*	*POL (64) LITH (37)*
**OTHER IN EUROPE**		**25**	**120**	**103**	**340**
***Non-EU FMC *^§^,**				*53*	*107*
	*mainly from*			*SWZ (46)*	*ICE (95)*
***Rest of Europe***				***50***	***233***
	*mainly from*			*RUS (15)*	*SERB (73) RUS (71)*
**NORTH AMERICA (US & CAN)**		**2**		**20**	**45**
**CENTRAL & SOUTH AMERICA**			**503**	**101**	
**OCEANIA**			**2**		**0**
**AFRICA**			**425**	**2,765**	**23**
**ASIA**		**19**	**10**	**519**	**234**
**NO SPECIFIED**		**0**	**0**	**8**	**87**

* Codes: AUS, Austria; BELA, Belarus; BELG, Belgium; CAN, Canada; CYP, Cyprus; CZE, Czech Republic; EU, European Union; EU 15, EU members before 2004; EU+12, EU new countries after 2004; FRA, France; GERM, Germany; GRE, Greece; HUN, Hungary; ICE, Iceland; I RE, Ireland; IT, Italy; LITH, Lithuania; NW, Norway; POL, Poland; PORT, Portugal; ROM, Romania; RUS, Russia (registered doctors from ex-USSR included); SERB, Serbia and Montenegro (registered doctors from ex-Yugoslavia included); SLK, Slovakia; SP, Spain; SWE, Sweden; SWZ, Switzerland; UK, United Kingdom; UKR, Ukraine.

† Foreign/foreign trained physicians (see text).

‡ "Free movement countries": non-EU countries with a contract on free movement of persons with EU: Iceland, Norway, Switzerland and Liechtenstein.

Table 2 shows data on European doctors practising abroad, either within the EU or elsewhere. Ireland is the European country with the highest percentage of doctors practising abroad, 47.5%, with Malta a distant second (in both cases the majority of their doctors are registered in the UK). In the case of countries that recently joined the EU, Malta, Romania, Hungary and Poland show figures higher than 5% for medical professionals practising abroad; for non-member states, all of the EEA countries outside the EU (Iceland, Switzerland and Norway), Serbia-Montenegro and Albania show that levels.

**Table 2:** Emigration factor for the European countries (European source-country perspective)

		**Physicians registered in other countries**		
			
**European countries**	Physicians in the country*	**Total**	**Only in other European countries**	**Emigration factor**^†^
**EU-15**				
Ireland	11,141	10,065	6,019	47.5%
Luxembourg	1,206	263	263	17.9%
Greece	47,944	5,804	4,090	10.8%
Austria	27,413	2,373	1,631	8.0%
Belgium	46,268	3,936	2,519	7.8%
United Kingdom	133,641	8,824	1,708	6.2%
Spain	135,300	8,530	3,960	5.9%
Netherlands	50,854	3,103	2,250	5.8%
Germany	277,885	14,196	9,734	4.9%
Sweden	29,122	1,446	1,094	4.7%
Denmark	15,653	774	616	4.7%
Italy	241,000	8,758	3,778	3.5%
Finland	16,446	357	269	2.1%
France	203,487	3,945	2,197	1.9%
Portugal	34,440	358	199	1.0%
**EU+12**				
Malta	1,254	376	328	23.1%
Romania	42,538	4,397	1,523	9.4%
Hungary	32,877	2,461	1,043	7.0%
Poland	95,272	6,568	3,130	6.4%
Slovakia	17,172	888	888	4.9%
Czech Republic	35,960	1,809	900	4.8%
Bulgaria	28,128	1,084	545	3.7%
Cyprus	1,864	48	48	2.5%
Latvia	6,940	172	129	2.4%
Lithuania	13,682	338	274	2.4%
Estonia	6,118	92	77	1.5%
Slovenla	4,475	44	44	1.0%
**Non-EU FMC**				
Iceland	1,056	318	137	23.1%
Switzerland	25,921	3,085	1,101	10.6%
Norway	14,200	788	711	5.3%
**Other European countries**				
Serbia and Mont.^‡^	21,738	2,808	1,367	11.4%
Albania	4,100	390	325	8.7%
Bosnia & Herzegovina	5,576	113	113	2.0%
Croatia	10,820	176	176	1.6%
Russian Federation^§^	609,043	7,099	2,039	1.2%
Ukraine	143,202	1,017	1,017	0.7%
Macedonia	4,459	10	10	0.2%
Belarus	45,027	76	76	0.2%
Moldova	11,246	5	5	0.0%
Georgia	20,962	5	5	0.0%
Uzbekistan	71,623	12	12	0.0%

*: Number of physicians in the respective country. Source: World Health Organization^23^.

^† ^Emigration factor: the relation between national (national-trained) doctors practising abroad and the total number of doctors related to that country -practising in it or abroad-

^‡^: Includes physicians registered as from ex-Yugoslavia

^§^:Include physicians registered as from ex-USSR

Other explanations in footnote on Table 1.

The number of professionals emigrating after the year 2004 to Germany, France and Norway have maintained previous trends (Table 3).

**Table 3:** Average annual variation in the number of foreign EEA physicians working in Germany, France and Norway between 2003 and 2005.

	**Years**	
**Countries**	**2003**	**2004–2005**
**European Union**		
Austria	168	157
Belgium	102	92
**Czech Republic**	52	33
**Cyprus**	3	-2
Denmark	31	-5
**Estonia**	5	3
Finland	3	-10
France	0	7
Germany	86	78
Greece	86	114
**Hungary**	12	23
Italy	88	89
**Latvia**	1	3
**Lithuania**	17	13
Luxembourg	17	2
**Malta**	0	1
Netherlands	9	14
**Poland**	196	160
Portugal	6	9
Ireland	2	3
**Slovakia**	74	74
**Slovenia**	3	6
Spain	33	30
Sweden	-3	-61
United Kingdom	23	15
**Non-EU FMC**		
Iceland	1	-10
Norway	1	2
Switzerland	8	8

In bold, EU countries joined to EU in 2004.

Source: elaborated through data from the German^15^, French^18 ^and Norwegian^19 ^medical associations.

The main results of the qualitative evaluations received from those medical organisations responding to the survey were as follows:

• **Sweden: **15% of the approximately 32,000 licensed doctors are foreigners, primarily from other Nordic countries, from within the EU or from Asia. Some 400 foreign doctors were registered in the last year and it is estimated that some 400 Swedish professionals are practising in other countries, principally in the US and Norway.

• **Hungary: **8% of the approximately 36,000 licensed doctors (these figures include dentists) are from other countries, principally Hungarian-speaking minorities of Romania, the Ukraine and Serbia, with a smaller presence of doctors from Slovakia. A significant number of Hungarian doctors are practising abroad, principally in the UK, the US, Germany and Austria. Last year, over a thousand doctors requested information about the documentation necessary to practise in other countries of the European Union while there are an estimated 2,400 vacancies for doctors in the country.

• **Austria: **the number of foreign doctors may be underestimated in our study since a large number have acquired Austrian citizenship in recent years.

## Discussion

The data available from different countries presents certain limitations, such as the lack of uniformity in registers and definitions [24]. In making an analysis according to countries of origin, we also must take in account that information from some countries of destination is either non-existent, outdated (most European information dates from 2001 or before) or fragmentary, except for those countries whose data were newly obtained in this research.

Previous studies revealed the existence of four poles of attraction and exchange of professionals: the UK and Ireland, the Nordic countries, Germany-Austria and France-Belgium-Luxembourg [12,13]. Our data confirm the existence of those poles, although for Ireland it is difficult to draw conclusions as up to a third of registered doctors reside outside of the country [20]; data for United Kingdom could be affected, although in a lesser extent, by the same reason [24]. Switzerland, with 19·1% of doctors of foreign origin, appears also as an important destination for professionals.

There are different profiles for foreign doctors in various EU countries. While in Austria, Norway, Portugal and France more than half of all foreign doctors are from other EU countries, three quarters of foreign doctors in the UK, and possibly in Ireland, are from outside the EU, generally developing countries, a situation which may give rise to ethical questions [2,25]. Conditions in Hungary and Poland are clearly different, with most foreign doctors being from neighbouring countries.

The origin of doctors is a key question in evaluating the impact professional emigration can have on different countries. A large percentage of Irish doctors, some 47.5%, practice in other countries, especially the UK and the USA. It should be noted that medical training in Ireland is financed to a large extent through the payments of students from outside the European Union, which explains the high number of places reserved for these students [2,26]. Countries whose registers show the country where training was received (as in the case of the UK and the US) offer figures for Ireland which are far higher than those they would offer if registering doctors with Irish nationality.

The high number of professionals from Malta and Poland practising abroad is cause for concern among health authorities in both countries [9,27]. The influence of the EU on bordering states in Eastern Europe is difficult to establish and may be underestimated in our study. It is probable that part of the emigration from these countries is directed, as indicated by data from Poland and Hungary, to the newest member states which have a limited representation in our study.

The information available for emigration in recent years does not indicate an effect of EU enlargement in the exodus of doctors from new member states to Norway, France, and Germany. However, it should be noted that these countries still maintained strict controls over the circulation of workers from the new member states during the period analysed. The recent study by the European Commission evaluating the impact of temporary measures governing the free movement of workers adopted by some member states in the wake of the 2004 EU expansion also failed to detect any significant evidence of increased migratory patterns [28], nor differences between countries where restrictions were applied and where there were not [12]. Nevertheless, physicians' migration is a growing phenomenon in Europe, as table 3 shows.

For the purposes of this study, temporal information was unavailable for the UK. Figures published in other places for 2002 are extrapolations from the National Health Service census and it is therefore difficult to compare these figures with those of registered doctors in the General Medical Council for 2005. While taking these considerations into account, significant increases have been seen in doctors licensed in Poland (x4.7), Greece, Italia (x3.2 in both cases) and Germany (x2.6), although we cannot determine the impact of the EU expansion since no figures are available between 2002 and 2005. Poland is currently experiencing a significant emigration of people to the UK (some 0.69% of its population has registered to work in the UK in the last two years while these figures are surpassed by those for Lithuania, Estonia and Slovakia), and accounted for 62% of health workers from new EU member states registered in the UK during this period [29].

## Conclusion

The increasing mobility of physicians within the European Union should be a cause for consideration for those planning health care services both at a European level and within member states [10,30]. While complete information is lacking, the existing data does provide a valuable basis for analysis and professional and international organisations are currently taking steps to update and present their information in this issue [15-19,24]. Data retrieved in this article show varying profiles in migration figures between European countries, with different countries being strong importers and exporters of physicians. Moving towards self-sufficiency in medical care personnel may prove to be an effective way to ensure health care services while deterring the migration of professionals from those countries that most need their expertise [11].

## Competing interests

There isn't any external source of financing or support, so author's work has been independent from any financial pressures. All authors declare that they have no competing interests.

## Authors' contributions

MAG participated in design, data collection, analysis and discussion of results and writing of the successive texts, including the final version, and as the first author of the article, accepts full responsibility for the conduct of the research; CA participated in the discussion of study design and in the analysis of data and results; AO participated in the analysis of data and results. All the authors have contributed substantially to the discussion and the final version of the manuscript, and have seen and approved its final version.

## Pre-publication history

The pre-publication history for this paper can be accessed here:


